# Commensal Bacteria and Expression of Two Major Intestinal Chemokines, TECK/CCL25 and MEC/CCL28, and Their Receptors

**DOI:** 10.1371/journal.pone.0000677

**Published:** 2007-07-25

**Authors:** François Meurens, Mustapha Berri, Richard H. Siggers, Benjamin P. Willing, Henri Salmon, Andrew G. Van Kessel, Volker Gerdts

**Affiliations:** 1 Lymphocyte et Immunité des Muqueuses, UR 1282, Infectiologie Animale et Santé Publique, Institut National de la Recherche Agronomique, Nouzilly, France; 2 Department of Animal and Poultry Science, University of Saskatchewan, Saskatoon, Saskatchewan, Canada; 3 Vaccine and Infectious Disease Organization, University of Saskatchewan, Saskatoon, Saskatchewan, Canada; Centre de Recherche Public-Santé, Luxembourg

## Abstract

**Background:**

CCL25/TECK and CCL28/MEC are CC chemokines primarily expressed in thymic dendritic cells and mucosal epithelial cells. Their receptors, CCR9 and CCR10, are mainly expressed on T and B lymphocytes. In human, mouse, pig and sheep CCL25 and CCL28 play an important role in the segregation and the compartmentalization of the mucosal immune system. As evidenced by early comparisons of germ-free and conventional animals, the intestinal bacterial microflora has a marked effect on host intestinal immune functions. However, little is known about the impact of bacterial colonization on constitutive and induced chemokine expressions as well as on the generation of anti-inflammatory mechanisms.

**Methodology/Principal Findings:**

Therefore, we decided to focus by qPCR on the mRNA expression of two main gut chemokines, CCL25 and CCL28, their receptors CCR9 and CCR10, the Tregs marker Foxp3 and anti-inflammatory cytokines TGF-β and IL-10 following colonization with different bacterial species within the small intestine. To accomplish this we used an original germ-free neonatal pig model and monoassociated pigs with a representative Gram-negative (*Escherichia coli*) or Gram-positive (*Lactobacillus fermentum*) commensal bacteria commonly isolated from the neonatal pig intestine. Our results show a consistent and marked effect of microbial colonization on the mRNA expression of intestinal chemokines, chemokine receptors, Foxp3 and TGF-β. Moreover, as evidenced by *in vitro* experiments using two different cell lines, the pattern of regulation of CCL25 and CCL28 expression in the gut appears complex and suggests an additional role for *in vivo* factors.

**Conclusions/Significance:**

Taken together, the results highlight the key role of bacterial microflora in the development of a functional intestinal immune system in an elegant and relevant model for human immune system development.

## Introduction

For a species to be a relevant model of human disease, it must simulate human conditions in both disease and health. Over the years, swine have been one of the most frequently used model resembling humans in almost all aspects analyzed. They are similar in size, feeding patterns, immune system, skin structure, renal, cardiac and pulmonary physiology and anatomy to man [1], [2].

Chemokines are involved in inflammation regulation, leukocyte trafficking, embryogenesis, hematopoiesis, and immune cell differentiation [3], [4]. They are highly basic heparin-binding proteins of 70–125 amino acids with molecular masses ranging from 6 to 14 kDa which exert their functions by binding specific seven-transmembrane G-protein-coupled receptors [5], [6]. The CC chemokine ligand 25 (CCL25) was first described in mouse and human thymus and is also known as thymus-expressed chemokine (TECK) [7]. In the mouse, the pig and the sheep, CCL25 is constitutively expressed by epithelial cells in the intestine and by dendritic cells in the thymus. CCL25 is involved in recruitment of T-precursor cells to fetal thymi and lymphocyte homing to the small intestine (SI) [7]–[16]. Lymphocyte recruitment to the gastrointestinal tract is mediated by a variety of interactions including integrin α4β7, which interacts with mucosal addressin cellular adhesion molecule-1 (MadCAM-1) expressed on endothelial cells in the gut lamina propria (LP) and in the gut-associated lymphoid tissues [17], [18]. In addition, chemokine receptor 9 (CCR9), the main receptor for CCL25, is specifically expressed on a subset of gut-homing T cells expressing integrin α4β7, as well as on IgA-secreting cells from gastrointestinal organs [8], [9], [15], [16], [19], [20]. CCL28, also called mucosae-associated epithelial chemokine (MEC), is expressed in most human, mouse, pig and sheep mucosal tissues including the salivary gland, mammary gland, small and large intestines and trachea, where it appears to be predominantly produced by epithelial cells [12], [13], [21], [22]. Cells expressing CCR10 are mainly IgA and IgM plasmablasts and some T lymphocytes [22]–[26]. These cells are thought to migrate in response to CCL28 and α4β1^high^/vascular cell adhesion molecule-1 interaction [22]–[26]. Hence, both CCL25 and CCL28 play essential roles in intestinal homing of immunoglobulin A antibody secreting cells (IgA-ASCs) and T cells and with their respective receptors serve to segregate and compartmentalize the mucosal immune system with important implications for the development of effective vaccines [27]–[29].

The gastrointestinal tract of an adult pig contains approximately 10^14^ prokaryotic and eukaryotic microorganisms [30]. This immense load of commensal bacteria means that the number of bacterial cells within the intestine is greater than the total number of eukaryotic cells within the host. As evidenced by early comparisons of germ-free and conventional animals, this population of bacteria has a marked effect on host intestinal physiology including morphology, mucus secretion, nutrient digestion and metabolism and immune function [31]–[34]. Hitherto, very little data is available about the effect of bacterial colonization on the constitutive or induced expression of chemokines. Recently, a study in mice has demonstrated an unique pattern of regulation for CCL25 expression [35]. It appeared that CCL25 transcription was independent of the members of the LT/TNF family of cytokines, which are known to control the constitutive expression of homeostatic chemokines in the SI, as well as inflammatory stimuli and proinflammatory chemokines [35]. These results are surprising since it was previously shown that TNF-α could increase the expression of CCL25 in SI murine LP [36]. Moreover, constitutive CCL25 mRNA expression has been shown to be independent of the presence of intestinal bacteria and lymphocytes [35]. The study suggested the involvement of Caudal-related homeobox transcription factors (Cdx), only expressed *in vivo*, in maintaining the high and tissue selective expression of CCL25 in the murine SI epithelium [35]. Unlike CCL25, CCL28 expression is better understood and is clearly controlled, at least for the induced expression, by inflammatory stimuli in murine and human airway epithelial cells through an NFκB-dependent mechanism [37]–[39].

In order to bring new insights into the complex interactions between the immune system and the SI commensal flora, we decided to focus on the mRNA expression of the two main gut chemokines, CCL25 and CCL28, and their receptors following colonization with different bacterial species along the length of the SI. To accomplish this, we used a germ-free neonatal pig model and monoassociated pigs with a representative Gram-negative (*Escherichia coli*) or Gram-positive (*Lactobacillus fermentum*) commensal bacteria commonly isolated from the neonatal pig intestine. Our results showed a consistent and marked effect of microbial colonization on intestinal chemokines and chemokine receptor expression. This effect depends of the bacterial strain. Moreover, as evidenced by *in vitro* experiments, the pattern of CCL25 and CCL28 expression regulation in the gut appears complex and suggests an additional role for *in vivo* factors.

## Results

### Expression of CCL25, CCL28, CCR9 and CCR10 transcripts in segments collected at 5–95% of small intestine length in gnotobiotic and conventional pigs

CCL25 and CCL28 play a crucial role in lymphocyte trafficking to the gut. Since little is known about their interactions with the gut commensal flora, we decided to assess mRNA expression of CCL25, CCL28, CCR9 and CCR10 by qPCR in segments collected at 5–95% of SI length in gnotobiotic and conventional pigs.

**Figure 1:**
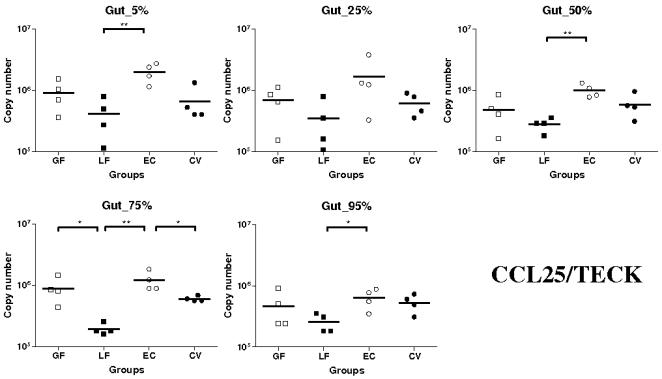
Expression of CCL25/TECK mRNA in segments collected at 5–95% (pyloric sphincter = 5%; ileo-cecal junction = 95%) of SI length in gnotobiotic and conventional pigs derived by caesarean section and reared in isolators until 13 days of age. cDNA was synthesized from 50 ng of RNA and subjected to qPCR using primer/probe sets for CCL25/TECK. Levels of expression are expressed in copy number. Copy numbers and median from four animals are shown in each group (GF: germ free; LF: *Lactobacillus fermentum*; EC: *Escherichia coli*; CV: conventionalized with fresh adult porcine fecal material). *, *P*<0.05, **, *P*<0.01 (Student's *t* test and Wilcoxon Signed Rank Test (Exact)).

The highest levels of CCL25 mRNA were observed in the EC gnotobiotic group (Fig. 1). At the 5, 50, 75 and 95% small intestinal (SI) locations, CCL25 mRNA expression was higher in the EC than in the LF groups (*P*<0.01 except at 95% SI location where it was <0.05). Similarly, at 75% SI location, more CCL25 transcripts were observed in the EC than in the conventional pig (CV) groups (*P*<0.05) (Fig. 1). Additionally, the median expression of CCL25 mRNA in LF group was lower than in the germ free (GF) group. However these observations were not statistically significant except at the 75% SI location (*P*<0.05). The median expression in GF and CV groups were not significantly different (Fig. 1).

**Figure 2:**
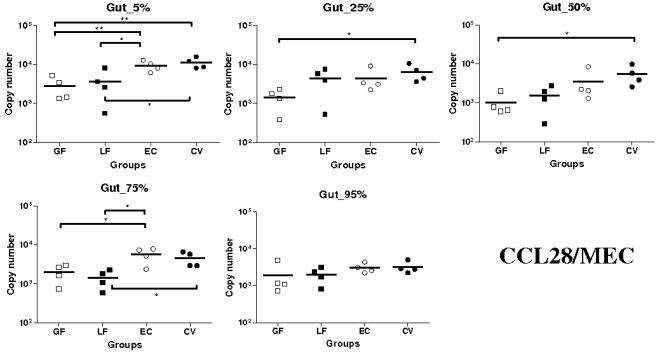
Expression of CCL28/MEC mRNA in segments collected at 5–95% (pyloric sphincter = 5%; ileo-cecal junction = 95%) of SI length in gnotobiotic and conventional pigs derived by caesarean section and reared in isolators until 13 days of age. cDNA was synthesized from 50 ng of RNA and subjected to qPCR using primer/probe sets for CCL28/MEC. Levels of expression are expressed in copy number. Copy numbers and median from four animals are shown in each group (GF: germ free; LF: *Lactobacillus fermentum*; EC: *Escherichia coli*; CV: conventionalized with fresh adult porcine fecal material). *, *P*<0.05, **, *P*<0.01 (Student's *t* test and Wilcoxon Signed Rank Test (Exact)).

Unlike CCL25, the level of CCL28 mRNA expression was generally low in the gut, especially in the GF and LF groups (Fig. 2). The highest levels of expression were observed in the CV and EC groups. Significantly more CCL28 transcripts were detected in the CV than in the GF groups at 5, 25, and 50% SI locations (*P*<0.05 except at 5% SI location where it was <0.01). Similarly, at 5 and 75% SI locations, CCL28 mRNA was more numerous in the EC than in GF groups (*P*<0.01 at 5% and <0.05 at 75% SI locations) (Fig. 2). Moreover, at 5% and 75% SI location, CCL28 levels of expression were significantly higher in CV and EC than LF groups (*P*<0.05).

**Figure 3:**
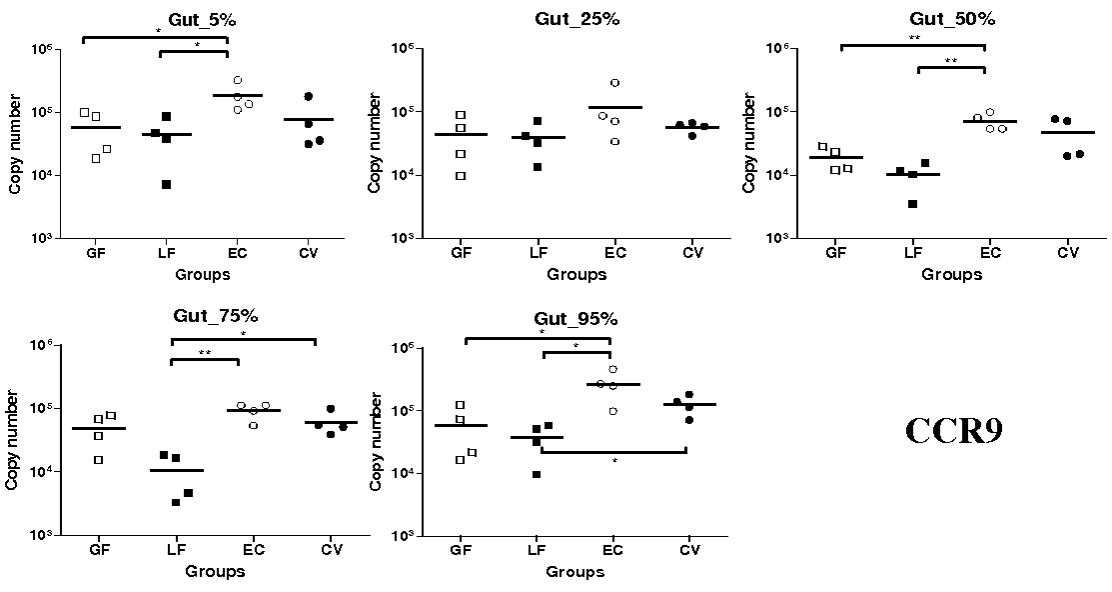
Expression of CCR9 mRNA in segments collected at 5–95% (pyloric sphincter = 5%; ileo-cecal junction = 95%) of SI length in gnotobiotic and conventional pigs derived by caesarean section and reared in isolators until 13 days of age. cDNA was synthesized from 50 ng of RNA and subjected to qPCR using primer/probe sets for CCR9. Levels of expression are expressed in copy number. Copy numbers and median from four animals are shown in each group (GF: germ free; LF: *Lactobacillus fermentum*; EC: *Escherichia coli*; CV: conventionalized with fresh adult porcine fecal material). *, *P*<0.05, **, *P*<0.01 (Student's *t* test and Wilcoxon Signed Rank Test (Exact)).

CCR9 mRNA pattern of expression was very similar to what we observed for CCL25 (Fig. 3). Indeed, the highest levels of expression for this receptor were observed in the EC group. Levels of expression of CCR9 mRNA were significantly higher in the EC than in the LF groups at 5, 50, 75 and 95% SI locations (*P*<0.05 at 5 and 95% SI locations and <0.01 at 50 and 75% SI locations). Furthermore, at 5, 50 and 95% SI locations, CCR9 mRNA was significantly more expressed in the EC than in the GF groups (*P*<0.05 except at 50% SI location where it was <0.01) (Fig. 3). As observed in the EC group, we detected higher CCR9 mRNA levels of expression in the CV than in the LF groups at 75 and 95% SI locations (*P*<0.05).

**Figure 4:**
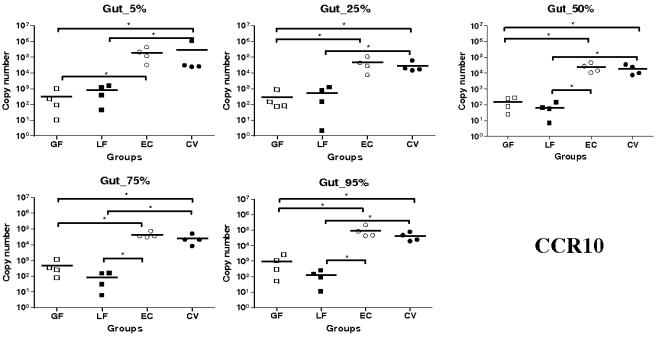
Expression of CCR10 mRNA in segments collected at 5–95% (pyloric sphincter = 5%; ileo-cecal junction = 95%) of SI length in gnotobiotic and conventional pigs derived by caesarean section and reared in isolators until 13 days of age. cDNA was synthesized from 50 ng of RNA and subjected to qPCR using primer/probe sets for CCR10. Levels of expression are expressed in copy number. Copy numbers and median from four animals are shown in each group (GF: germ free; LF: *Lactobacillus fermentum*; EC: *Escherichia coli*; CV: conventionalized with fresh adult porcine fecal material). *, *P*<0.05, **, *P*<0.01 (Student's *t* test and Wilcoxon Signed Rank Test (Exact)).

For CCR10 mRNA expression, a marked difference was observed between the EC and CV groups and the LF and GF groups (Fig. 4). CCR10 transcripts were barely detectable in the GF and LF groups while they were highly expressed in the EC and CV groups. Then, the levels of expression of CCR10 mRNA were significantly higher in the EC and CV than in the GF groups at all the SI locations (*P*<0.05). Similarly, levels of expression of CCR10 mRNA were generally higher in CV and EC groups than in the LF group (*P*<0.05) (Fig. 4).

In summary, CCL25 and CCR9 mRNA were highly expressed in the EC group; significantly more than in the LF group for both and the GF group for CCR9. Levels of expression of CCL28 and CCR10 transcripts were higher in the EC and CV than in the other groups (except at 95% SI location for CCL28). This observation is particularly marked for CCR10.

### Expression of CCL25, CCL28, CCR9 and CCR10 transcripts along the small intestine in *E. coli* monoassiociated and conventional pigs

**Figure 5:**
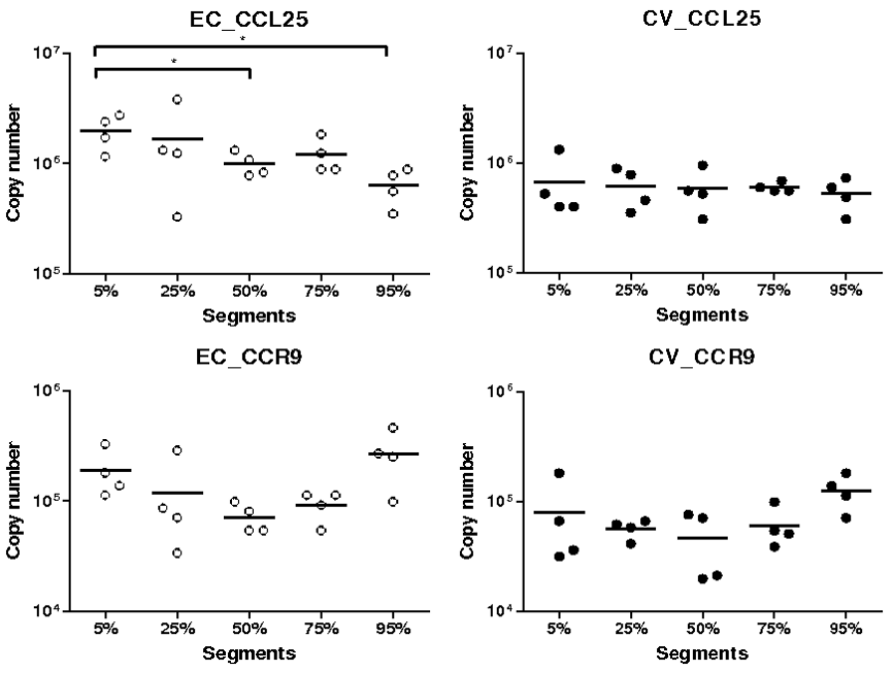
Comparison of the expression of CCL25/TECK and CCR9 mRNA in *Escherichia coli* (EC) gnotobiotic and conventional (CV) pigs in segments collected at 5–95% (pyloric sphincter = 5%; ileo-cecal junction = 95%) of SI length. cDNA was synthesized from 50 ng of RNA and subjected to qPCR using primer/probe sets for CCL25/TECK and CCR9. Levels of expression are expressed in copy number. Copy numbers and median from four animals are shown in each group. *, *P*<0.05, **, *P*<0.01 (Student's *t* test and Wilcoxon Signed Rank Test (Exact)).

**Figure 6:**
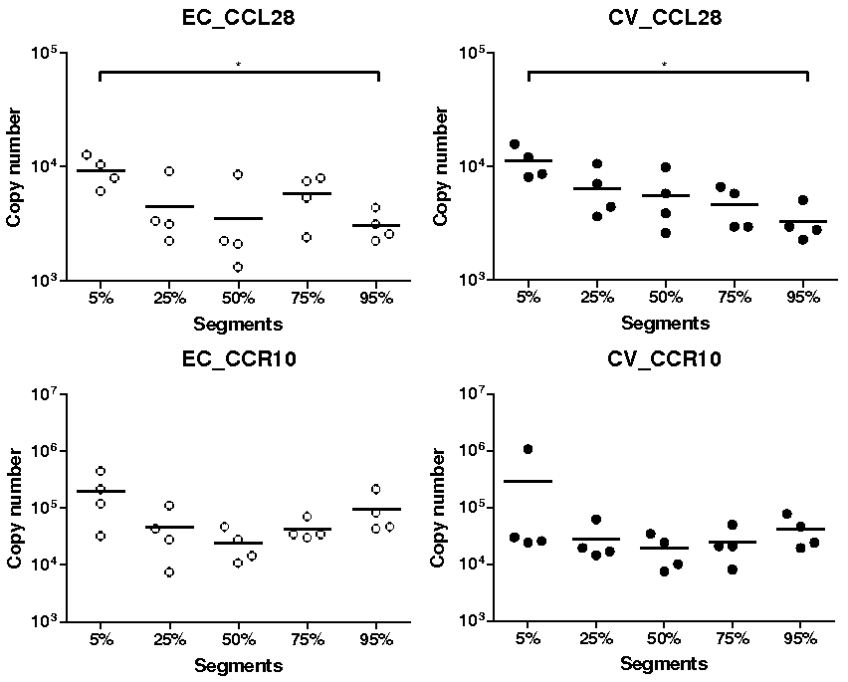
Comparison of the expression of CCL28/MEC and CCR10 mRNA in *Escherichia coli* (EC) gnotobiotic and conventional (CV) pigs in segments collected at 5–95% (pyloric sphincter = 5%; ileo-cecal junction = 95%) of SI length. cDNA was synthesized from 50 ng of RNA and subjected to qPCR using primer/probe sets for CCL28/MEC and CCR10. Levels of expression are expressed in copy number. Copy numbers and median from four animals are shown in each. *, *P*<0.05, **, *P*<0.01 (Student's *t* test and Wilcoxon Signed Rank Test (Exact)).

The levels of expressions of CCL25 mRNA were similar in all different locations (Fig. 5). Nevertheless, we observed higher levels of expression in the proximal SI compared to more distal SI in the EC group (*P*<0.05). For its receptor, mRNA expression was not different between locations in both groups, EC and CV (Fig. 5). CCL28 and CCR10 mRNA levels of expression were generally not significantly different between locations (Fig. 6). However higher expression of CCL28 mRNA was observed at 5 than at 95% SI locations in both groups (*P*<0.05).

In summary, within the SI, location had only slight influences on the expression of chemokines CCL25 and CCL28 and their receptors.

### Expression of Foxp3, IL-10 and TGF-β transcripts in segments collected at 5–95% of small intestine length in gnotobiotic and conventional pigs

**Figure 7:**
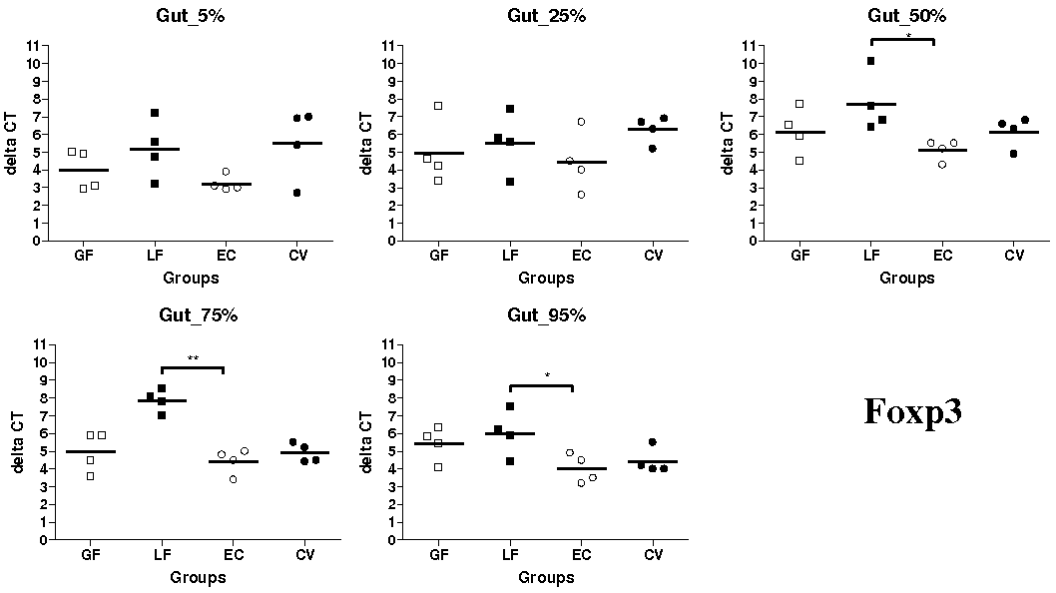
Comparison of the expression of Foxp3 mRNA in segments collected at 5–95% (pyloric sphincter = 5%; ileo-cecal junction = 95%) of SI length in gnotobiotic and conventional pigs derived by caesarean section and reared in isolators until 13 days of age. cDNA was synthesized from 50 ng of RNA and subjected to qPCR using primer/probe sets for Foxp3 and GAPDH. Delta CT was calculated as follows: Foxp3 CT – GAPDH CT. Foxp3 quantities and median are shown for four animals in each group (GF: germ free; LF: *Lactobacillus fermentum*; EC: *Escherichia coli*; CV: conventionalized with fresh adult porcine fecal material), with samples expressing the highest levels of Foxp3 mRNA demonstrating the lowest delta CT value. *, *P*<0.05, **, *P*<0.01 (Student's *t* test and Wilcoxon Signed Rank Test (Exact)).

Regulatory T cells (Tregs) suppress tissue damage that occurs as a consequence of inflammatory or antimicrobial immune responses. The transcription factor Foxp3, known to inhibit several cytokine genes both in human and mouse, is considered as a good general marker of Tregs [40], [41]. The function of Tregs relies on their ability to produce IL-10 and surface-bound TGF-β. Recently a population of CCR10-expressing Tregs was identified in inflamed human liver [39]. Because Tregs can play an important role in the relation of the immune system with the commensal flora, Tregs marker Foxp3 and Tregs products TGF-β and IL-10 were studied by qPCR in gnotobiotic and conventional pigs at the different locations of the SI. The highest levels of expression of Foxp3 were detected in the EC group (Fig. 7). Foxp3 mRNA expression was generally higher in the EC group *vs* GF, LF and CV groups. Nevertheless, the differences between groups were mostly not significant (*P*>0.05). Foxp3 mRNA levels of expression were significantly higher in EC group than in LF group at 50, 75 and 95% SI locations (*P*<0.05 at 50 and 95% SI locations and <0.01 at 75% location) (Fig. 7). Regarding IL-10, mRNA levels of expression were very low, in both EC and CV groups, or undetectable, in both GF and LF groups (data not shown). TGF-β mRNA were usually more expressed in EC group, statistically more than in the LF (*P*<0.05) and CV (*P*<0.01) groups at 75% SI locations, and less expressed in the LF and CV groups (data not shown). Nevertheless, given to the important variations observed between animals, these results have to be analyzed cautiously.

### CCL25 and CCL28 mRNA expression is not enhanced by inducers of inflammatory chemokines *in vitro*

Since we observed an influence of bacteria on the expression of CCL25 and CCL28 mRNA, we decided to analyse *in vitro* the expression of CCL25 and CCL28 in the IPI-2I porcine intestinal cell line; SJPL porcine lung cell line served as a control. To determine whether CCL25 and CCL28 mRNA levels were enhanced after exposure to inflammatory stimuli, confluent cells were stimulated with various concentrations of 055:B5 LPS (100 and 5000 ng/ml), IL-1β (10 and 1000 ng/ml) and TNF-α (50 and 1000 ng/ml) during 6 h. These molecules failed to enhance CCL25 and CCL28 mRNA levels (data not shown). Similarly, addition of the bacteria themselves (MOI of 10 CFU/cell with EC, LF, and a 1∶1 mix of both) to the cells for 6 h failed to induce an increase in the expression of CCL25 and CCL28 mRNA (data not shown). These results may suggest a complex regulation of CCL25 and CCL28 mRNA expression.

**Figure 8:**
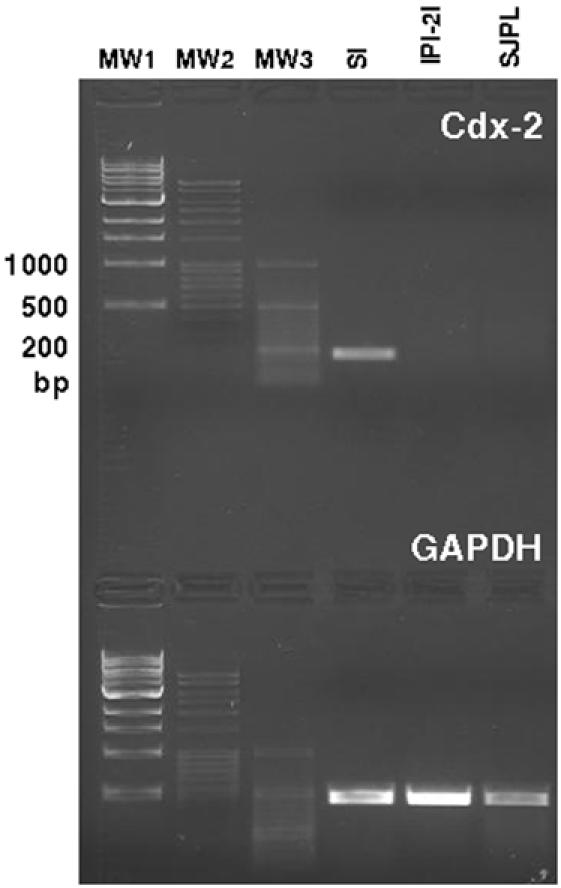
Expression of Cdx-2 and GAPDH mRNA by conventional PCR amplifications. cDNA was synthesized from 50 ng of RNA extracted from small intestine (SI), IPI-2I, and SJPL cells. After 30 cycles of amplification, the PCR products were run on a 1.5% agarose gel and stained with ethidium bromide. Three DNA molecular weight markers were used (MW1 from Amersham and MW2, MW3 from Fermentas). The PCR product sizes are presented in Table 1.

Because it has been recently shown that expression of Cdx transcription factors was correlated with the ability to express high levels of CCL25 mRNA [35], we decided to design primers for porcine Cdx2 (Cdx-1 EST was not available in pig) to assess its expression in both cell lines as well as in SI tissue. While SI cells expressed Cdx-2 mRNA, IPI-2I and SJPL cells failed to express Cdx-2 (Fig. 8). Consequently, in SI tissue, but not in IPI-2I or SJPL cells, Cdx-2 can bind to the putative binding motif within the TATA box of CCL25 promoter and consequently induce its expression.

**Table 1:** Primer sequences, annealing temperatures of primer sets (°C), and expected PCR fragment sizes (bp).

Primer Name	Primer sequence	Annealing temperature (°C) of primer set	cDNA PCR Product (bp)
CCL25 qPCR	(S) GCCTACCACAGCCACATTAAG	56	136
	(AS) GCTTCCCGCACACCATCTT		
CCL28 qPCR	(S) GCTGCTGCACTGAGGTTTC	56	145
	(AS) TGAGGGCTGACACAGATTC		
CCR9 qPCR	(S) CCAGATGACTACGGCTATGAC	56	150
	(AS) GGCACCCACGATGAACAC		
CCR10 qPCR	(S) GCCCGCAGAGCAGGTTTCC	60	136
	(AS) CAAAGAGACACTGGGTTGGAAG		
GAPDH qPCR	(S) CTCAACGGGAAGCTCACTGG	56	106
	(AS) TGATCTCATCATACTTGGCAGGTT		
Foxp3 qPCR	(S) GGTGCAGTCTCTGGAACAAC	63.3	148
	(AS) GGTGCCAGTGGCTACAATAC		
IL-10 qPCR	(S) ACCAGATGGGCGACTTGTTG	63	123
	(AS) TCTCTGCCTTCGGCATTACG		
TGF-β qPCR	(S) GGTTCTGGATCAGGCTTACC	57.1	101
	(AS) CGCCAAACCTCTCCAAATCG		
Cdx-2	(S) GGAACCTGTGCGAGTGGATG	65	168
	(AS) GCTCGGCCTTTCTCCGAATG		
GAPDH	(S) ACCACAGTCCATGCCATCAC	60	452
	(AS) TCCACCACCCTGTTGCTGTA		

## Discussion

As evidenced by early comparisons of germ-free and conventional animals, the intestinal population of bacteria has a marked effect on intestinal immune functions [32]–[34]. However, very little is known about the influence of commensal bacteria on CCL25 and CCL28 expression, two majors intestinal chemokines implicated in lymphocytes trafficking to the gut. In this study, taking advantage of a recently developed germ-free pig model and a previous study in pig [12], [33], we assessed mRNA expression of CCL25, CCL28, CCR9, CCR10, Foxp3, TGF-β and IL-10 at 5 regions corresponding to 5%, 25%, 50%, 75%, and 95% of the SI length following colonization with different bacterial species.

Concerning CCL25 mRNA expression in the gut, highest levels were observed in the EC group. However these levels were most often not statistically higher then those observed in the GF and CV groups suggesting, as observed in mice [35], a constitutive expression of CCL25 in the SI which is independent of commensal flora. Interestingly, we have however observed in all the segments a significantly higher expression of CCL25 mRNA in the EC group comparatively to the LF group. Moreover this expression in LF group was even significantly lower than in the GF group at 75% SI location. Taken together, these results may suggest a down-regulation of CCL25 in the presence of *Lactobacillus. fermentum* and an up-regulation in the presence of *Escherichia coli*. As evidenced in many recent studies probiotic bacteria like *Lactobacillus* or *Bifidobacteria* possess the ability to down-regulate the expression of some chemokines, for example interleukin-8 and interleukin-1β, and therefore they have the ability to decrease inflammation [42]–[46]. However, contrary to what we observed *in vivo*, we did not detect any up-regulation of CCL25 mRNA expression when intestinal epithelial cell line IPI-2I were stimulated with the bacteria used *in vivo* or TNF-α, IL-1β and 055:B5 LPS. Moreover, the constitutive expression of CCL25 mRNA in the cell lines was extremely low. Since it has been shown that Cdx transcription factors are implicated in CCL25 transcription [35], we further checked the expression of Cdx-2 in IPI-2I, SJPL lung cells and in SI tissue. Our results were in agreement with the previous study [35] and Cdx-2 transcripts were not detected in the porcine cell lines while there was an expression in the tissue suggesting the involvement of specific *in vivo* factors in the regulation pattern of CCL25. In summary, it appears that there is in the gut a constitutive expression of CCL25 independent of commensal flora and a potential induced expression, which can be linked to bacterial colonization to some extent, through complex pathway of regulation. Similarly to CCL25 mRNA, the highest levels of expression for CCR9 mRNA were observed in the EC group and the lowest in the LF group. These data, suggesting a potential increase in the migration of lymphocytes to the gut in the EC group, are consistent with the hypothesis of a down-regulation of CCL25 in the presence of *L. fermentum* and an up-regulation of the chemokine expression in the presence of *E. coli*. However, with the same animals [33], it has been shown that the number of proximal intestinal intra-epithelial lymphocytes (IELs) in conventionalized pigs was between 1.5 and 6.5-fold greater than in GF and monoassociated pigs [33]. This result is a little bit surprising since the highest CCR9 mRNA levels of expression were observed in the EC group suggesting a drastically higher expression of the receptor in this group. Nevertheless, because there is no available data about LP lymphocytes, a potential higher mRNA expression of CCR9 cannot be further confirmed. Contrary to what we observed with CCL25, there were no significant differences between SI locations in EC and CV groups. The lower expression of CCL25 mRNA in the last segment could be explained by the presence of Peyer's patches (PP) in this location since we have usually observed [12], lower expression of CCL25 in PP than in the rest of the SI. In the pig there are two types of PP: several separate patches in the jejunum and proximal ileum, and a long continuous patch in the distal ileum [1], [2].

Expression of CCL28 mRNA was generally higher in EC and CV groups with significant differences at 5, 25, 50 and 75% SI locations. Since it has been shown a clear relation between epithelial inflammation and CCL28 production [37]–[39], the higher expressions observed in EC and CV groups are probably caused by inflammation processes within piglets SI. In LF and GF groups, where there is no detectable inflammation, the CCL28 mRNA expression stayed very low. For CCR10, mRNA levels of expression were systematically higher in EC and CV groups with significant differences. Moreover, the mRNA expression was extremely low in the GF and LF groups. CCR10 is mainly expressed on IgA and IgM plasmablasts and some T lymphocytes [22]–[26]. Consequently very low CCR10 mRNA expression in GF and LF groups suggest a reduction in the migration of plasmablasts and lymphocytes to the gut even if there is evidence of a low expression of CCL28 mRNA in the tissue. As previously observed CCR10 mRNA expression does not strictly follow CCL28 mRNA expression [12]. CCL28 has two important roles in mucosal immunity; chemokine and antimicrobial peptide [47]. However, little is known in pig about the amount of CCL28 needed to attract lymphocytes and about the kinetic of lymphocyte trafficking to the gut. Furthermore it is difficult to assess the repartition of both CCL28 roles in a specific tissue. Taken together, we can postulate that there are both constitutive expression of CCL28 with mainly the antimicrobial activity and induced expression which is necessary for massive migration of lymphocytes to the gut. Identically to CCL25 and CCR9, we detected a significantly higher expression of CCL28 mRNA in the proximal segment and no statistically significant differences between segments for CCR10 mRNA expression. As postulated above, this observation could be explained by the presence of PP in the distal segments. Both with the lung and the intestinal epithelial cell lines, we failed to induce mRNA expression of CCL28 with TNF-α and IL-1β. This result is surprising with the lung epithelial cell line since previous studies in mouse and human have clearly shown an increase of CCL28 mRNA expression following stimulation of murine lung epithelial (MLE-12) and human airway epithelial-like cells (A549) [37], [38]. Potential explanations to this discrepancy could be the absence of some specific factors required for CCL28 expression in our cell line or some differences in the regulation of this chemokine between tissues and/or species.

Concerning the expression of Foxp3, it was generally higher in EC group than in the other groups, especially LF. This observation is not surprising since inflammation in LF and GF groups is very low or null meaning that there is less a need of Tregs in the SI of these piglets. It suggests, as shown in mice, that intestinal bacterial microflora plays a role in the generation of anti-inflammatory mechanisms in mucosal tissues [48], [49]. Indeed, recently a study has shown that bacterial antigens are crucial for the generation and/or expansion of Tregs [49]. Bacterial colonization of a healthy individual is of great importance to advise the immune system to generate regulatory mechanisms and maintain immunological homeostasis under normal conditions [49]. Because Tregs mediate suppression via TGF-β and/or IL-10, we further checked the mRNA expression of both cytokines and we observed generally higher expression in EC and CV groups than in the others. These results, consistent with previous observation in mice [49], suggest again a key role of intestinal bacterial microflora, especially Gram-negative bacteria, in the generation of Tregs.

In summary, we have shown that the mRNA expression of CCL25 and CCL28 is clearly affected by intestinal bacterial microflora, up-regulated or down-regulated depending of the strain, with consecutive impact on lymphocyte migration as strongly suggested by mRNA expression of CCR9 and CCR10. Moreover, it seems that bacterial colonization is also of great importance in the generation of Tregs since expression of Foxp3, IL-10 and TGF-β was different between groups. Then, as suggested by cellular experiments, the pattern of regulation of CCL25 and CCL28 in the gut appears complex and suggests a role for *in vivo* factors. Taken together, the results highlight the role of a diversified population of bacteria in the expression of intestinal chemokines and the development of a competent immune system.

## Materials and Methods

### Experimental design

Germ-free pigs were derived by caesarean section and reared in sterilized HEPA-filtered isolator units as previously described [33]. A total of 16 pigs (Large White X White Duroc) were aseptically reared in one of four isolator units. Pig assignment to the isolators was balanced for litter of origin and sex. One isolator was maintained as germ-free (GF). Two isolators were designated as monoassociated; pigs in 1 isolator were orally inoculated with Gram-positive *Lactobacillus fermentum* (LF) and in the other with a non-pathogenic Gram-negative *Escherichia coli* (EC). Pigs in the fourth isolator were used as a control and orally inoculated with fresh fecal material from adult pigs (CV). LF and EC inoculants were isolated from the caecum of a healthy adult pig. Both organisms were cultured for 18 h at 37°C in a tryptic soy broth (BBL, Sparks, MD) and a sub sample from each culture was taken for enumeration. Fresh feces were obtained from conventionally reared pigs at Prairie Swine Centre Inc. (Saskatoon, SK, Canada) and mixed 1∶1 with sterile phosphate-buffered saline (0.01 M phosphate, 0.15 M NaCl, pH 7.4). Sealed tubes containing the appropriate 18 h bacterial culture or feces were aseptically passed into the appropriate isolator. At 24 h and 30 h post-partum, the pigs were orally inoculated with 2 ml of their respective bacterial culture by adding the inoculants to the milk prior to bottle feeding. Pigs in the conventional isolator received 6 ml of inoculate (2 ml of each of the monoassociated inoculants and 2 ml of the fecal slurry at each feeding). Viable cell counts in the LF and EC inoculants were 10^8^ colony forming units (CFU)/ml and 10^9^ CFU/ml, respectively. Microbial status of pigs was confirmed at the end of the study as described [33]. Experimental protocols were approved by the Animal Care Committee of the University of Saskatchewan and were performed in accordance with recommendations of the Canadian Council on Animal Care (1993).

### Tissue collection

Pigs were euthanized by CO_2_ asphyxiation, weighed and exsanguinated on day 13. An incision was made along the ventral midline of the abdomen to remove the intestinal tract and the SI was dissected from the mesentery. The length of the SI was measured and regions corresponding to 5, 25, 50, 75, and 95% in length beginning at the pyloric sphincter were identified. Two 10 cm-segments at each region were excised, rinsed with cold physiological saline (Bimeda-MTC, Cambridge, Canada), blotted dry on paper, weighed, snap frozen in liquid nitrogen and stored at −80°C for mRNA analysis.

### Messenger RNA expression analysis using real-time PCR

Whole intestinal tissue was subjected to mechanical disruption under liquid nitrogen by using a mortar and pestle. Total RNA was extracted from 20 to 30 mg of tissue using the RNeasy® Mini kit (Qiagen, Mississauga, Canada). Total RNA quantity and purity were determined by optical density (OD) at 260 and 280 nm wavelengths using a spectrophotometer (Ultrospec® 2000, Pharmacia Biotech, Baie d'Urfe, PQ). Then, RNA samples were treated with DNAse I Amp Grade (Invitrogen) (1 U/µg of RNA). The absence of genomic DNA contamination was validated by use of treated RNA as template directly in PCR. One microgram of total RNA was used for a reverse transcription reaction (total 21 µl) with Oligo(dt)_12–18_ primers and SuperScript^TM^ II reverse transcriptase (SuperScript^TM^ first strand synthesis system for RT-PCR, Invitrogen, Carlsbad, CA) for evaluation of relative expression. Negative controls were made by replacing the reverse transcriptase with diethyl pyrocarbonate-treated water. The resulting single-stranded cDNA was then used in for real-time PCR (qPCR) analysis (iCycler iQ Real-Time PCR detection system, Bio-Rad, Hercules, CA) for evaluation of relative expression. cDNA was combined with primer/probe sets and IQ SYBR Green Supermix (Bio Rad) according to the manufacturer's recommendations. All primers were designed using Clone Manager (Scientific & Educational Software, Cary, NC) (Table 1) and were purchased from Invitrogen. Primers for Foxp3, interleukin-10 (IL-10) and transforming growth factor-beta (TGF-β) were based on porcine GenBank sequences AY669812, NM_214041 and NM_001038639, respectively. The PCR conditions were 95°C for 3 min, followed by 45 cycles with denaturation at 95°C for 15 s, annealing temperature (Table 1) for 30 s, and elongation at 72°C for 30 s. The specificity of the PCR reactions was assessed by the analysis of the melting curves of the products, size verification and sequencing of the amplicons. To normalize the amount of cDNA, we sampled equal tissue sizes, quantitated RNA, assessed its quality prior to reverse transcription, and used a reference gene. Samples were normalized internally using the average cycle threshold (CT) of glyceraldehyde-3-phosphate dehydrogenase (GAPDH) as a reference in each tissue. The suitability of GAPDH as a reference was confirmed by the lack of variation observed between animals from a same group. To quantify the numbers of copies, plasmids containing chemokine or chemokine receptor cDNA were linearized with *Xho*I (New England Biolabs) and ten two-fold dilutions of each plasmid (1 ng/ml) were used to create a standard curve for quantitation of the RNA-generated cDNA [12]. Values were expressed as transcript copy number per 50 ng of total input RNA, which were determined in each sample by interpolation with the respective standard curves. The correlation coefficients of the CCL25, CCL28, CCR9, CCR10, Foxp3, IL-10, and TGF-β standard curves were 0.996, 1, 0.996, 0.998, 0.994, 0.997, and 1 respectively, and the concentration of the test samples were calculated from the standard curves, according to the formula *y* = −M*Ct+*B*, where M is the slope of the curve, *Ct* the point during the exponential phase of amplification in which the fluorescent signal is first recorded as being statistically significant above background, and *B* the y-axis intercept. Only *Ct* values <40 were used for calculation of the PCR efficiency from the given slope according to the equation: PCR efficiency = (10^[−1/M]^−1)×100. All PCRs displayed efficiencies between 94% and 100%.

### Cell line culture

The porcine SI epithelial cell line IPI-2I (ECACC 93100622) was established from the ileum of an adult boar (SLA ^d/d^ haplotype) and immortalized by transfection with an SV40 plasmid (pSV3-neo) [50]. IPI-2I cells were maintained in DMEM (Invitrogen Life Technologies) supplemented with 10% FCS (Sigma-Aldrich), 4 mM L-glutamine (Invitrogen Life Technologies), insulin 10 ug/ml (Sigma-Aldrich), 50 U/ml penicillin and 50 mg/ml streptomycin (Invitrogen Life Technologies). The porcine epithelial-like cell line (St. Jude porcine lung [SJPL] cells; ATCC PTA-3256) was spontaneously established from the normal lungs of a normal 4-week-old female Yorkshire at St. Jude Children's Research Hospital [51]. SJPL cells were maintained in DMEM (Invitrogen Life Technologies) supplemented with 10% fetal bovine serum, 1% sodium pyruvate, 1% L-glutamine, 1.4% MEM nonessential amino acids, and 1% antibiotic-antimycotic solution (Sigma-Aldrich). In all experiments cells were used between passages 30 and 70. Cells were allowed to adhere to the matrix of the culture plate overnight, before 6 h stimulation with IL-1β (10 and 1000 ng/ml) (Biosource International), TNF-α (50 and 1000 ng/ml) (Biosource International) or LPS 055:B5 (100 and 5000 ng/ml) (Cambrex Bio Science). Total RNA was then isolated from these cells for use in qPCR as described above. All stimulations were carried out in triplicate.

### Incubation of intestinal cells with bacteria

IPI-2I cells were grown to confluency in 6-well plates and incubated with 10 CFU/cell (EC, LF, and a mix 1∶1 of both bacteria) for 6 h in triplicate. After incubation, cells were washed twice and then the total RNA was isolated from the cells for use in qPCR as described above.

### Caudal-related homeobox transfection factor 2 conventional polymerase chain reaction

BLAST searches of the GenBank ESTs database and Pig EST data explorer, using known sequences of human Cdx-1 and -2 identified expressed sequence tags (EST) for porcine Cdx-2 ( GenBank accession no. CK458871). Primers were designed using Clone Manager (Scientific & Educational Software, Cary, NC) (Table 1) and were purchased from Invitrogen. Samples were normalized using GAPDH as a reference in sample. cDNAs from the cells were amplified with 0.5 U Phusion polymerase (Finnzymes, Espoo, Finland), 0.2 mM of each dNTP (Finnzymes), 0.5 µM sense and antisense primers (Table 1) and 1X Phusion HF buffer (Finnzymes). The amplification conditions were carefully chosen for Cdx-2 and GAPDH genes to obtain signals in the linear amplification range and consisted of a first denaturation at 98°C for 30 s, followed by 30 cycles [with denaturation at 98°C for 10 s, annealing temperature at 60°C (GAPDH) and 65°C (Cdx-2) for 20 s, and elongation at 72°C for 20 to 30 s] and a final extension at 72°C for 8 min. Amplification products (10 µl of each) were separated by electrophoresis in a 1% TBE agarose (Sigma) gel stained with ethidium bromide and visualized on a UV transilluminator.

### Statistical analysis

Data for the comparison of differences in mRNA expression between tissues and pigs are expressed as copy numbers or delta CT. After logarithmic transformation, most of obtained data were normally distributed as confirmed by Shapiro-Wilk normality test (using Statistix 7.0®, Analytical software, Tallahassee, FL, USA). When the data were independent and normally distributed, group means were compared using Student's t-test. Paired, normally distributed data were analysed using Student's Paired t-test (using GraphPad Prism® software version 4.00, GraphPad Software Inc., San Diego, CA, USA). Independent, non-normally distributed data were analysed using the Wilcoxon Rank Sum Test (Exact) and paired, non-normally distributed data were analysed using the Wilcoxon Signed Rank Test (Exact). Differences between groups were considered significant when *P<*0.05. Only the most relevant comparisons are presented in figures and in the text.

## References

[1] Pastoret P-P, Griebel P, Bazin H, Govaerts A, Pastoret P-P, Griebel P, Bazin H, Govaerts A (1998). Immunology of the pig.. Handbook of vertebrate immunology.

[2] Tumbleson M, Schook LB, Tumbleson M, Schook LB (1996). Advances in swine in biomedical research.. Advances in swine in biomedical research.

[3] Charo IF, Ransohoff RM (2006). The many roles of chemokines and chemokine receptors in inflammation.. N Engl J Med.

[4] Olson TS, Ley K (2002). Chemokines and chemokine receptors in leukocyte trafficking.. Am J Physiol Regul Integr Comp Physiol.

[5] Kim CH, Broxmeyer HE (1999). Chemokines: signal lamps for trafficking of T and B cells for development and effector function.. J Leukoc Biol.

[6] Stein JV, Nombela-Arrieta C (2005). Chemokine control of lymphocyte trafficking: a general overview.. Immunology.

[7] Vicari AP, Figueroa DJ, Hedrick JA, Foster JS, Singh KP (1997). TECK: a novel CC chemokine specifically expressed by thymic dendritic cells and potentially involved in T cell development.. Immunity.

[8] Choe H, Farzan M, Konkel M, Martin K, Sun Y (1998). The orphan seven-transmembrane receptor apj supports the entry of primary T-cell-line-tropic and dualtropic human immunodeficiency virus type 1.. J Virol.

[9] Kunkel EJ, Campbell JJ, Haraldsen G, Pan J, Boisvert J (2000). Lymphocyte CC chemokine receptor 9 and epithelial thymus-expressed chemokine (TECK) expression distinguish the small intestinal immune compartment: Epithelial expression of tissue-specific chemokines as an organizing principle in regional immunity.. J Exp Med.

[10] Liu C, Saito F, Liu Z, Lei Y, Uehara S (2006). Coordination between CCR7- and CCR9-mediated chemokine signals in pre-vascular fetal thymus colonization.. Blood.

[11] Liu C, Ueno T, Kuse S, Saito F, Nitta T (2005). The role of CCL21 in recruitment of T-precursor cells to fetal thymi.. Blood.

[12] Meurens F, Berri M, Whale J, Dybvig T, Strom S (2006). Expression of TECK/CCL25 and MEC/CCL28 chemokines and their respective receptors CCR9 and CCR10 in porcine mucosal tissues.. Vet Immunol Immunopathol.

[13] Meurens F, Whale J, Brownlie R, Dybvig T, Thompson DR (2007). Expression of mucosal chemokines TECK/CCL25 and MEC/CCL28 during fetal development of the ovine mucosal immune system.. Immunology.

[14] Wurbel MA, Philippe JM, Nguyen C, Victorero G, Freeman T (2000). The chemokine TECK is expressed by thymic and intestinal epithelial cells and attracts double- and single-positive thymocytes expressing the TECK receptor CCR9.. Eur J Immunol.

[15] Zaballos A, Gutierrez J, Varona R, Ardavin C, Marquez G (1999). Cutting edge: identification of the orphan chemokine receptor GPR-9-6 as CCR9, the receptor for the chemokine TECK.. J Immunol.

[16] Zabel BA, Agace WW, Campbell JJ, Heath HM, Parent D (1999). Human G protein-coupled receptor GPR-9-6/CC chemokine receptor 9 is selectively expressed on intestinal homing T lymphocytes, mucosal lymphocytes, and thymocytes and is required for thymus-expressed chemokine-mediated chemotaxis.. J Exp Med.

[17] Hu MC, Crowe DT, Weissman IL, Holzmann B (1992). Cloning and expression of mouse integrin beta p(beta 7): a functional role in Peyer's patch-specific lymphocyte homing.. Proc Natl Acad Sci U S A.

[18] Berlin C, Berg EL, Briskin MJ, Andrew DP, Kilshaw PJ (1993). Alpha 4 beta 7 integrin mediates lymphocyte binding to the mucosal vascular addressin MAdCAM-1.. Cell.

[19] Pabst O, Ohl L, Wendland M, Wurbel MA, Kremmer E (2004). Chemokine receptor CCR9 contributes to the localization of plasma cells to the small intestine.. J Exp Med.

[20] Norment AM, Bogatzki LY, Gantner BN, Bevan MJ (2000). Murine CCR9, a chemokine receptor for thymus-expressed chemokine that is up-regulated following pre-TCR signaling.. J Immunol.

[21] Pan J, Kunkel EJ, Gosslar U, Lazarus N, Langdon P (2000). A novel chemokine ligand for CCR10 and CCR3 expressed by epithelial cells in mucosal tissues.. J Immunol.

[22] Wang W, Soto H, Oldham ER, Buchanan ME, Homey B (2000). Identification of a novel chemokine (CCL28), which binds CCR10 (GPR2).. J Biol Chem.

[23] Kunkel EJ, Kim CH, Lazarus NH, Vierra MA, Soler D (2003). CCR10 expression is a common feature of circulating and mucosal epithelial tissue IgA Ab-secreting cells.. J Clin Invest.

[24] Bonini JA, Steiner DF (1997). Molecular cloning and expression of a novel rat CC-chemokine receptor (rCCR10rR) that binds MCP-1 and MIP-1beta with high affinity.. DNA Cell Biol.

[25] Jaimes MC, Rojas OL, Kunkel EJ, Lazarus NH, Soler D (2004). Maturation and trafficking markers on rotavirus-specific B cells during acute infection and convalescence in children.. J Virol.

[26] Lazarus NH, Kunkel EJ, Johnston B, Wilson E, Youngman KR (2003). A common mucosal chemokine (mucosae-associated epithelial chemokine/CCL28) selectively attracts IgA plasmablasts.. J Immunol.

[27] Feng N, Jaimes MC, Lazarus NH, Monak D, Zhang C (2006). Redundant role of chemokines CCL25/TECK and CCL28/MEC in IgA+ plasmablast recruitment to the intestinal lamina propria after rotavirus infection.. J Immunol.

[28] Kunkel EJ, Campbell DJ, Butcher EC (2003). Chemokines in lymphocyte trafficking and intestinal immunity.. Microcirculation.

[29] Kunkel EJ, Butcher EC (2003). Plasma-cell homing.. Nat Rev Immunol.

[30] Savage DC (1977). Microbial ecology of the gastrointestinal tract.. Annu Rev Microbiol.

[31] Corthesy B, Gaskins HR, Mercenier A (2007). Cross-Talk between Probiotic Bacteria and the Host Immune System.. J Nutr.

[32] Furuse M, Okumura J (1994). Nutritional and physiological characteristics in germ-free chickens.. Comp Biochem Physiol A Physiol.

[33] Shirkey TW, Siggers RH, Goldade BG, Marshall JK, Drew MD (2006). Effects of commensal bacteria on intestinal morphology and expression of proinflammatory cytokines in the gnotobiotic pig.. Exp Biol Med (Maywood).

[34] Wostmann BS, Wostmann BS (1996). Germfree and gnotobiotic animal models: background and applications.. Germfree and gnotobiotic animal models: background and applications.

[35] Ericsson A, Svensson M, Arya A, Agace WW (2004). CCL25/CCR9 promotes the induction and function of CD103 on intestinal intraepithelial lymphocytes.. Eur J Immunol.

[36] Hosoe N, Miura S, Watanabe C, Tsuzuki Y, Hokari R (2004). Demonstration of functional role of TECK/CCL25 in T lymphocyte-endothelium interaction in inflamed and uninflamed intestinal mucosa.. Am J Physiol Gastrointest Liver Physiol.

[37] O'Gorman MT, Jatoi NA, Lane SJ, Mahon BP (2005). IL-1beta and TNF-alpha induce increased expression of CCL28 by airway epithelial cells via an NFkappaB-dependent pathway.. Cell Immunol.

[38] English K, Brady C, Corcoran P, Cassidy JP, Mahon BP (2006). Inflammation of the respiratory tract is associated with CCL28 and CCR10 expression in a murine model of allergic asthma.. Immunol Lett.

[39] Eksteen B, Miles A, Curbishley SM, Tselepis C, Grant AJ (2006). Epithelial inflammation is associated with CCL28 production and the recruitment of regulatory T cells expressing CCR10.. J Immunol.

[40] Sakaguchi S (2005). Naturally arising Foxp3-expressing CD25+CD4+ regulatory T cells in immunological tolerance to self and non-self.. Nat Immunol.

[41] Banham AH, Powrie FM, Suri-Payer E (2006). FOXP3+ regulatory T cells: Current controversies and future perspectives.. Eur J Immunol.

[42] Zhang L, Li N, Caicedo R, Neu J (2005). Alive and dead Lactobacillus rhamnosus GG decrease tumor necrosis factor-alpha-induced interleukin-8 production in Caco-2 cells.. J Nutr.

[43] Riedel CU, Foata F, Philippe D, Adolfsson O, Eikmanns BJ (2006). Anti-inflammatory effects of bifidobacteria by inhibition of LPS-induced NF-kappaB activation.. World J Gastroenterol.

[44] O'Hara AM, O'Regan P, Fanning A, O'Mahony C, Macsharry J (2006). Functional modulation of human intestinal epithelial cell responses by Bifidobacterium infantis and Lactobacillus salivarius.. Immunology.

[45] Lammers KM, Vergopoulos A, Babel N, Gionchetti P, Rizzello F (2005). Probiotic therapy in the prevention of pouchitis onset: decreased interleukin-1beta, interleukin-8, and interferon-gamma gene expression.. Inflamm Bowel Dis.

[46] Petrof EO, Kojima K, Ropeleski MJ, Musch MW, Tao Y (2004). Probiotics inhibit nuclear factor-kappaB and induce heat shock proteins in colonic epithelial cells through proteasome inhibition.. Gastroenterology.

[47] Hieshima K, Ohtani H, Shibano M, Izawa D, Nakayama T (2003). CCL28 has dual roles in mucosal immunity as a chemokine with broad-spectrum antimicrobial activity.. J Immunol.

[48] Waidmann M, Allemand Y, Lehmann J, di Genaro S, Bucheler N (2002). Microflora reactive IL-10 producing regulatory T cells are present in the colon of IL-2 deficient mice but lack efficacious inhibition of IFN-gamma and TNF-alpha production.. Gut.

[49] Strauch UG, Obermeier F, Grunwald N, Gurster S, Dunger N (2005). Influence of intestinal bacteria on induction of regulatory T cells: lessons from a transfer model of colitis.. Gut.

[50] Kaeffer B, Bottreau E, Velge P, Pardon P (1993). Epithelioid and fibroblastic cell lines derived from the ileum of an adult histocompatible miniature boar (d/d haplotype) and immortalized by SV40 plasmid.. Eur J Cell Biol.

[51] Seo SH, Collisson EW (1997). Specific cytotoxic T lymphocytes are involved in in vivo clearance of infectious bronchitis virus.. J Virol.

